# Promising prognostic value of ATP binding cassette transporters and their correlation with tumor-infiltrating immune cells in lung adenocarcinoma

**DOI:** 10.1016/j.gendis.2023.101099

**Published:** 2023-09-14

**Authors:** Linzi Wei, Qian Yu, Xueying Yang

**Affiliations:** Department of Thoracic Surgery, The Fourth Affiliated Hospital of China Medical University, Shenyang, Liaoning 110032, China

Lung cancer (LC) is a prevalent and fatal malignancy, with high incidence and mortality rates.^1^ ATP binding cassette (ABC) transporters, a superfamily of integral membrane proteins, use ATP hydrolysis to facilitate substrate transport across cellular membranes.^2^ The pivotal role of ABC transporters in tumorigenesis was initially established by Mochida,^3^ who demonstrated that the loss of ABCB1 suppresses intestinal polyp formation and tumor progression in mice. Recent investigations have reported correlations between ABC transporters and the tumor microenvironment and cancer immunotherapy response, suggesting their modulatory effects on tumor progression and immunotherapy.^4^^,^^5^ However, at present, there is a lack of comprehensive and complete analytical studies on the expression, prognosis, and immune status of all genes in the ABC transporter family. In this study, we utilized public databases, coupled with quantitative real-time PCR and immunohistochemistry verification in lung adenocarcinoma (LUAD) cell lines and tissue specimens, to identify the consistent expression of ABC transporters and PD-L1 at both mRNA and protein levels. Our findings provide novel insights into potential therapeutic targets and prognostic biomarkers for LUAD.

In order to investigate the differential expression of ABC transporters comprehensively and systematically in LUAD, RNA-seq data from Oncomine databases were utilized to analyze the differential mRNA expression of ABCs (Fig. 1A; Fig. S1A–C). Compared with adjacent non-cancerous tissues, 20 of the 43 genes (45%) in the ABC gene family showed high expression in cancer tissues, and 6 genes (7%, 6/43) displayed low expression.

**Figure 1:**
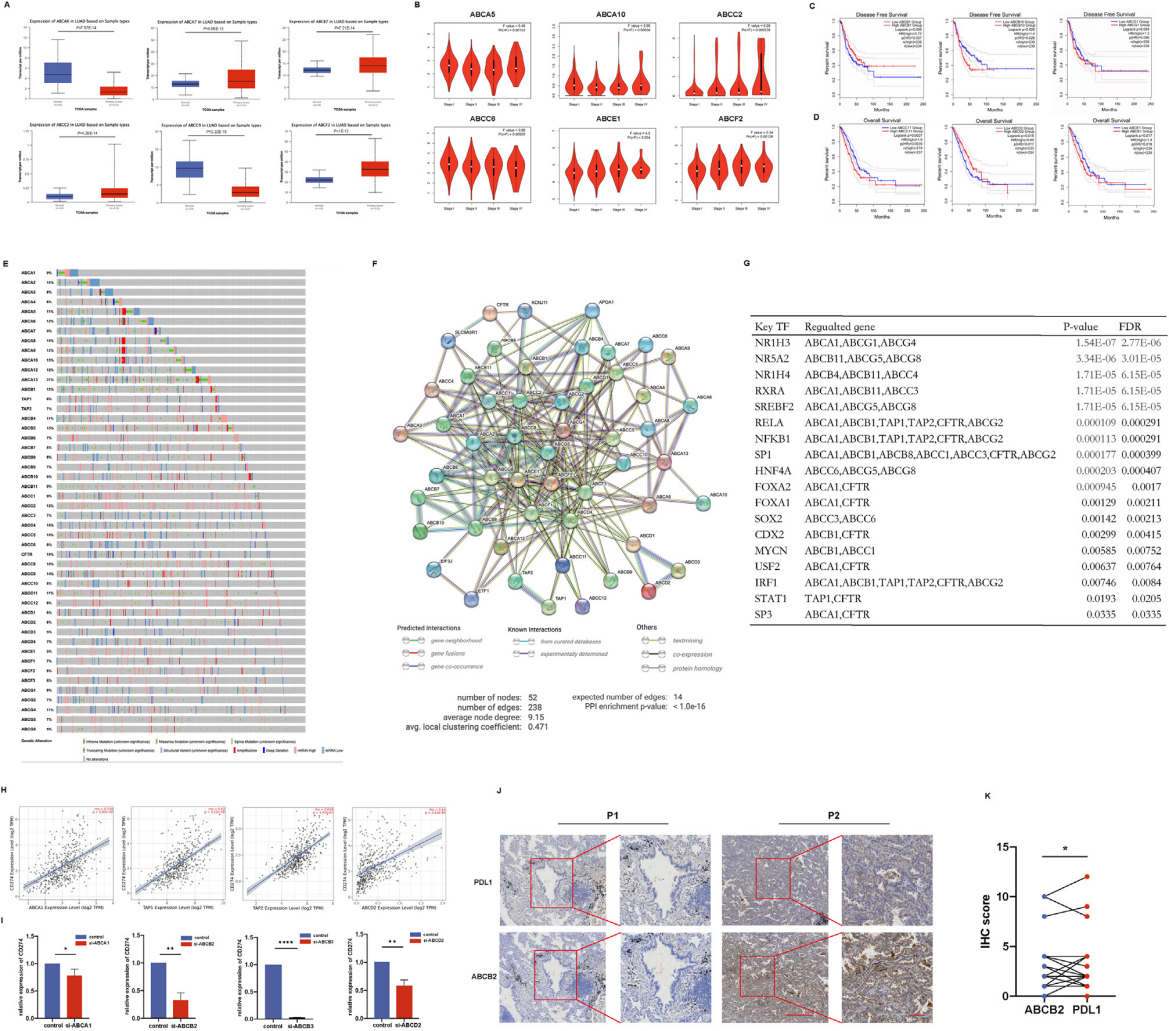
The ABC transporters' expression, prognostic value, and impact on PD-L1 expression in lung adenocarcinoma (LUAD). **(A)** Bioinformatic analysis showed the mRNA expression of ABCs in LUAD (cancer *vs*. paired para-cancer tissue). **(B)** The expression of ABCs at different clinical stages in patients with LUAD. **(C, D)** Prognostic value of ABC transporters. OS and DFS were plotted as Kaplan–Meier curves. ^∗^*P* < 0.05. **(E)** Genetic and epigenetic alterations of ABC transporters in LUAD. **(F)** Protein interactions analyzed on the STRING website. The line indicates the predicted mode of molecular action. **(G)** Key transcriptional factors targeting ABCs. **(H)** Spearman correlation analysis of ABCs and PD-L1 according to the TIMER2.0 platform. **(I)** The altered expression of PD-L1 upon ABC knockdown. ^∗^*P* < 0.05. **(J)** Representative images of immunohistochemistry staining for PD-L1 and ABCB2 in LUAD tissues. Scale bars, 100 μm. **(K)** The relationship between PD-L1 and ABCB2 at the protein level. The Spearman analysis showed an immunohistochemistry score correlation between PD-L1 and ABCB2 (*R* = 0.439, *P* = 0.015).

Using the GEPIA 2 platform, our analysis unveiled a significant correlation between the expression levels of *ABCA5*, *ABCA8*, *ABCA10*, *ABCC2*, *ABCC4*, *ABCC6*, *ABCC12*, *ABCE1*, and ABCF2, and pathological stage (Fig. 1B; Fig. S2). Furthermore, our prognosis analyses indicated that elevated levels of ABCB1, ABCB10, and ABCG1 were notably linked with poor disease-free survival (Fig. 1C; Fig. S3), while elevated levels of ABCA3, ABCC8, ABCC11, ABCC12, ABCD2, and ABCE1 were notably linked with poor overall survival (Fig. 1D; Fig. S4).

Using the cBioPortal, our investigation uncovered remarkable alterations in ABC transporters among 92% of 503 LUAD patients. Notably, the mutation rate of ABCB1 was found to be as high as 21% for these patients. Enhanced mRNA expression and amplification were the most observed changes in these samples (Fig. 1E).

To investigate the protein interaction network, gene family function, and transcriptional regulatory mechanisms involved in ABC transporters, we have refined our study as follows. Using the STRING database (Fig. 1F), we generated a protein–protein interaction network of ABC transporters and computationally analyzed potential functional associations with APOA1, EIF3J, SLC9A3R1, KCNJ11, and ETF1. An analysis of Gene Ontology enrichment and KEGG pathway analyses was carried out to investigate ABC transporters, and the top terms are presented (Fig. S5). Potential transcription factor targeting ABC transporters was predicted with TRRUST v2. Eighteen distinct transcription factors are involved in regulating ABC transporters (Fig. 1G).

We analyzed the correlation between ABC expression and various tumor-infiltrating immune cells using the TIMER online tool. Our analysis highlighted positive and negative correlations between ABC transporter expression and immune cell infiltration. For example, the expression of *ABCB1*, *ABCB2*, and *ABCB3* showed a positive correlation with CD8^+^ T cell and CD4^+^ T cell infiltration, while ABCB6 showed a negative correlation with CD8^+^ T cell infiltration.

We identified obvious expression correlation between *CD274* and *ABCA1* (*R* = 0.539, *P* = 3.83e-40), *ABCB2* (*R* = 0.63, *P* = 3.13e-58), *ABCB3* (*R* = 0.648, *P* = 1.43e-62), and *ABCD2* (*R* = 0.54, *P* = 2.63e-40) (Fig. 1H; Fig. S7). To validate the predicted results, we generated siRNAs targeting these ABCs and investigated their impact on *PD-L1* expression. Our results demonstrated that mRNA expression of *PD-L1* was significantly reduced following the knockdown of *ABCA1*, *ABCB2*, *ABCB3*, and *ABCD2* (Fig. 1I). Subsequently, we conducted immunohistochemistry analysis to examine the protein expression in 30 LUAD cases (Table S1–4). ABCB2 was identified as the sole gene that exhibits expression correlation with PD-L1 at both mRNA and protein levels (Fig. 1J, K; Fig. S8A, B).

In our investigation, we explored the expression and mutations of ABC transporters in LUAD, as well as their potential associations with immune cell infiltration and PD-L1 expression. Interestingly, we observed a consistent correlation between ABCB2 and PD-L1 expression at both mRNA and protein levels, which could have promising implications for future anticancer treatments.

## Ethics declaration

The collection of clinical cancer patient specimens was approved by the Ethical Committee of the Fourth Affiliated Hospital of China Medical University.

## Conflict of interests

The authors declare no conflict of interests.

## Funding

This research was supported by The Basic Scientific Research Projects of Colleges and Universities of Liaoning Province, China (No. LFWK201713). The authors are responsible for the results and opinions provided by this research, and the sponsor is not responsible for the content published.
