# Supplementary material for: Promising prognostic value of ATP binding cassette transporters and their correlation with tumor-infiltrating immune cells in lung adenocarcinoma
Source: Genes Dis. 2023 Sep 14;11(5):101099. doi: 10.1016/j.gendis.2023.101099 (PMC11176628; doi:10.1016/j.gendis.2023.101099)
Supplement: Multimedia component 1 [file mmc1.docx]

**Materials and Methods**

**Oncomine**

Data regarding ABC mRNA expression in various cancer types were retrieved from the online database Oncomine. Differences in mRNA expression between cancer tissues and corresponding normal tissues were assessed using the following threshold parameters: a threshold p-value of 1E-4, fold-change > 2, and gene ranking in the top 10%. T test was performed to determine whether the difference in ABC expression was significant.

**UALCAN**

UALCAN, a comprehensive web portal, can be used to perform in-depth analyses of TCGA gene expression data. In our study, the differential expression data for ABC transporters between cancer tissues and normal tissues at the mRNA level were obtained using the “Expression Analysis” module of UALCAN and the “LUAD” dataset. T test was performed to analyze the difference in ABC expression, and P < 0.05 was considered to indicate statistical significance.

**GEPIA2**

Gene expression profiling interactive analysis (GEPIA2) is a newly developed interactive online platform for analyzing RNA sequencing data. It provides access to a large collection of data from The Cancer Genome Atlas (TCGA) and the Genotype-Tissue Expression (GTEx) project. In this study, we performed differential mRNA expression analysis (tumor vs. normal tissues), pathological stage analysis, and correlation analysis (ABC transporter expression vs. prognosis) with the “Single Gene Analysis” module ­­of GEPIA2. P < 0.05 was considered to indicate statistical significance.

**cBioPortal**

The cBioPortal for Cancer Genomics is an online open-access website resource for exploring, visualizing, and analyzing multidimensional cancer genomics data. Based on the TCGA and ICGA (International Cancer Genome Consortium) database, we investigated ABC predicted copy number alterations, mRNA expression, gene correlations and mutations.

**STRING**

The Search Tool for the Retrieval of Interacting Genes (STRING) is an online software of interactions of genes and proteins, aims to complement these with computational predictions of potential functions. We conducted a protein‒protein interaction (PPI) network analysis of differentially expressed ABCs to explore the interactions among them with STRING.

**DAVID 6.8**

DAVID 6.8, a web-based bioinformatics resource intended for functional genomics analysis, was used for annotation and visualization. In our study, Gene Ontology (GO) enrichment analysis (including the biological processes (BP), cellular components (CC), and molecular function (MF) categories) and Kyoto Encyclopedia of Genes and Genomes (KEGG) pathway enrichment analysis of the DEGs related to ABCs were performed using DAVID 6.8. The cutoff value for significant enrichment of GO terms and KEGG pathways was a false discovery rate (FDR) of <0.05.

**TRRUST v2**

TRRUST v2 is a database of reference TF–target regulatory interactions in humans based on literature curation. It contains information on 8444 transcription factor (TF)-target regulatory relationships for 800 human TFs and can provide information on how these interactions are regulated. In this study, we studied ABCs involved in transcriptional regulation in the context of human diseases.

**TIMER2.0**

TIMER2.0 is an intuitive tool to explore and visualize tumor immunologic and genomic data. In our study, we estimated the correlation between ABC expression and immune infiltration level (the abundance of six subgroups: B cells, CD4+ T cells, CD8+ T cells, macrophages, neutrophils, and dendritic cells) by applying a deconvolution method. In addition, we investigated the correlation between the expression levels of the immune checkpoint PD-L1 and ABCs. P < 0.05 was considered to indicate statistical significance.

**Cell Culture**

The A549 cell line, a commonly used lung cancer cell model derived from adenocarcinomic human alveolar basal epithelial cells, was grown in RPMI 1640 medium (Gibco, USA) containing 10% fetal bovine serum (Biological Industries, Israel), 100 units/ml penicillin and 100 µg/ml streptomycin and maintained at 37°C under 95% air and 5% CO2. The human lung carcinoma cell line A549 was obtained from ATCC and kept in the Research Center of the Fourth Affiliated Hospital of China Medical University.

**siRNA Transfection**

Tumor cells were transfected with different siRNAs using Lipofectamine 3000 Transfection Reagent (Invitrogen, USA) in RPMI 1640 serum-free medium. Commercially available nontargeting siRNA (si-Control) was used. All siRNAs were obtained from GenePharma (**Table. S1**). Six hours after transfection, the cells were cultured in serum-containing medium for a resting period of 24 h, and the cells were collected to extract total RNA.

**Quantitative Real-Time PCR**

Total RNA was extracted from cells in culture using TRIzol Reagent (Invitrogen, USA), homogenized in chloroform, and then purified by isopropanol and ethanol. For qPCR, RNA concentration and quality were assessed by a NanoPhotometer spectrophotometer (IMPLEN, Germany). At most, 1 μg of total RNA per sample was reverse transcribed with the PrimeScript™ RT Reagent Kit (TAKARA, Japan), and then real-time qPCR amplification was performed on a QuantStudio 3 instrument (Thermo, USA) with SYBR Premix Ex TaqTM (TAKARA, Japan). The gene expression level was normalized to GAPDH, and the relative expression of the gene was calculated by the 2−ΔΔCt method. Each reaction was set up with three replicates per group. All primer sequences in this study are included in **Table. S2**.

**Immunohistochemistry**

All tumor tissues used for immunohistochemical staining were excised from 30 patients hospitalized at the Fourth Affiliated Hospital of China Medical University. There were no age or gender limitations. To validate our results, we examined tumor tissue sections of surgically resected or biopsied samples from 30 patients with LUAD using IHC. Immunohistochemistry was performed using 5 μm tissue sections prepared from the best representative formalin-fixed paraffin-embedded blocks of lung adenocarcinoma cases, deparaffinized with xylene, and rehydrated with decreasing concentrations of ethanol in water. Slides were incubated with antibody overnight at 4°C. Next, slides were incubated with the secondary antibody for 30 min and DAB solution for 1 min. Images were captured on a Nikon microscope and analyzed using ImageJ. A total of 30 LUAD cases are listed in **Table. S3**. Antibody information is provided in **Table. S4**.

The extent of IHC staining was scored as 0-no staining (no visible difference from the control group), 1-weak staining (light yellow), 2-moderate staining (yellow) or 3-strong staining (dark yellow). The proportion of tumor cells in the sample was scored as 0-no positive cells, 1-1~25% positive cells, 2-26~50% positive cells, 3-51~75% positive cells, and 4-76~100% positive cells. The expression was evaluated by the extent score multiplied by the proportion score. A staining score of >7 was used to define high expression, and a score ≤7 was used to define low expression.

**Statistical analysis**

Statistical analysis was performed using GraphPad Prism 8 software. Each experiment was carried out at least in triplicate, and all results are presented as the mean ± SD. T tests were used to assess statistical significance. P <0.05 was considered to indicate statistical significance.

**Abbreviations**

BP Biological Process

CC Cellular Component

GO Gene Ontology

GSEA Gene Set Enrichment Analysis

ICGC International Cancer Genome Consortium

KEGG Kyoto Encyclopedia of Genes and Genomes

LUAD Lung Adenocarcinoma

MF Molecular Function

PPI Protein–Protein Interaction

TCGA The Cancer Genome Atlas

**Alternative Name**

ABCB2 TAP1

ABCB3 TAP2

ABCC7 CFTR

PD-1 PDCD1

PD-L1 CD274

**Fig. S1A.** This graphic was generated using Oncomine and indicates the numbers of datasets with statistically significant upregulation (red) or downregulation (blue) of ABC mRNA (cancer vs. corresponding normal tissue).

**Fig. S1B.**The GEPIA2 online tool was used to analyze ABC mRNA expression in LUAD. Red represents the level of mRNA expression the deeper the color, the higher the expression level.

**Fig. S1C.** mRNA expression of ABCs in LUAD (cancer vs. corresponding para-cancer tissue) by using the UALCAN online tool (TCGA database).

**Fig S2.** The expression of ABCs at different clinical stages (stage I, stage II, stage III, stage IV) in patients with LUAD.

**Fig S3.** Disease-free survival (DFS) of patients grouped based on the expression of ABCs in LUAD.

**Fig S4.** Overall survival (OS) of patients grouped based on the expression of ABCs in LUAD.

**Fig S5.** GO and KEGG enrichment analysis of ABC transporters in LUAD. The numbers below each panel are reference P values (−log10).

**Fig S6.** Correlation of ABC transporter mRNA expression with immune infiltration level using the TIMER2.0 online tool. ABC expression was associated with infiltration of CD8+ T cells, CD4+ T cells, B cells, neutrophils, macrophages, or DCs in LUAD.

**Fig S7.** Correlation of ABC transporter mRNA expression with PD-L1.

**Fig S8A.** Representative images of IHC staining for PD-L1, ABCA1, ABCB3 and ABCD2 in LUAD tissues. Scale bars, 100 μm;

**Fig S8B.** The relationship between PD-L1 and ABC transporters at the protein level. The Spearman statistical method showed correlations between PD-L1 and ABCA1 (R=0.304, P=0.103), ABCB3 (R=0.149, P=0.433), and ABCD2 (R=0.335, P=0.071).

| siRNA | sequence |
| --- | --- |
| \| ABCA1 \| \| --- \| \| ABCB2 \| \| ABCB3 \| \| ABCD2 \| | \| 5’-GCTGTGGAAGAACCTCACTTT-3’ \| \| --- \| \| 5’-CGGGATCTATAACAACACCAT-3’ \| \| 5’-CGGTTCTGTGAGGAACAACAT-3’ \| \| 5’-GCAGATCAGATGAACCTCATT-3’ \| |

Supplementary Table 1 siRNA Sequence

Supplementary Table 2 Primer Sequence

| Names | Primer’s sequence (5’-3’) | GC% | Tm(°C) | Length | Product size (bp) |
| --- | --- | --- | --- | --- | --- |
| PDL1 | F: GAATGCTGGTTCCCCTTTGC  R: GCAAGGGGTGGGGGATTAA | 55  55 | 59.75  59.96 | 20  20 | 261 |
| GAPDH | F:AAAAGGGCCCTGACAACTCT  R: TACATGACAAGGTGCGGCTC | 50  55 | 59.15  60.39 | 20  20 | 275 |

Supplemenatry Table 3 Patient Information

| Clinical information | Number of examples |
| --- | --- |
| Age  <60  ≥60 | 16  14 |
| Gender  Male  Female  T stage  T1  T2  T3  T4  N stage  N0  N1  N2  M stage  M0  M1  Clinical stage  I  II  III  IV | 15  15  25  3  0  2  23  5  2  29  1  22  4  3  1 |

| gene name | company | dilution ratio | species |
| --- | --- | --- | --- |
| \| PDL1 \| \| --- \| \| ABCA1 \| \| TAP1 \| \| TAP2 \| \| ABCD2 \| | \| Proteintech (66248-1-Ig) \| \| --- \| \| ABclonal （A16337） \| \| Proteintech (11114-1-AP) \| \| ABclonal （A1610） \| \| Abcam (ab238502) \| | 1:2500  1:200  1:600  1:1000  1:200 | \| mouse \| \| --- \| \| rabbbit \| \| rabbbit \| \| rabbbit \| \| rabbbit \| |

Supplemenatry Table 4 Antibody Information
