# Supplementary figures and images for: Promising prognostic value of ATP binding cassette transporters and their correlation with tumor-infiltrating immune cells in lung adenocarcinoma
Source: Genes Dis. 2023 Sep 14;11(5):101099. doi: 10.1016/j.gendis.2023.101099 (PMC11176628; doi:10.1016/j.gendis.2023.101099)

A

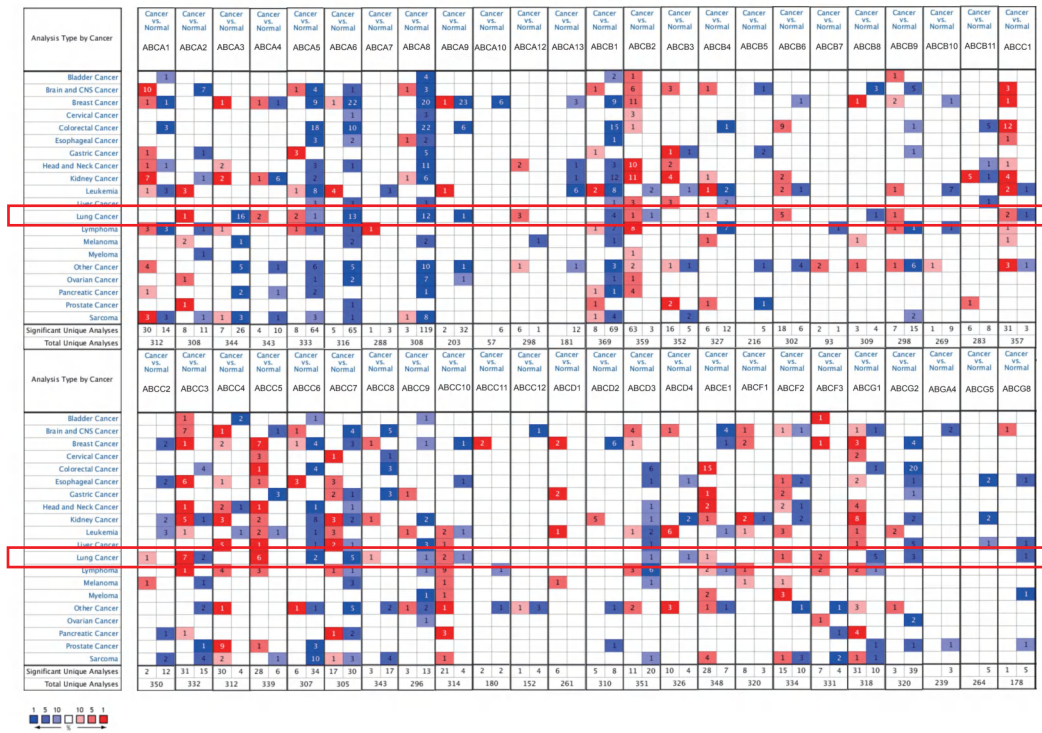

B

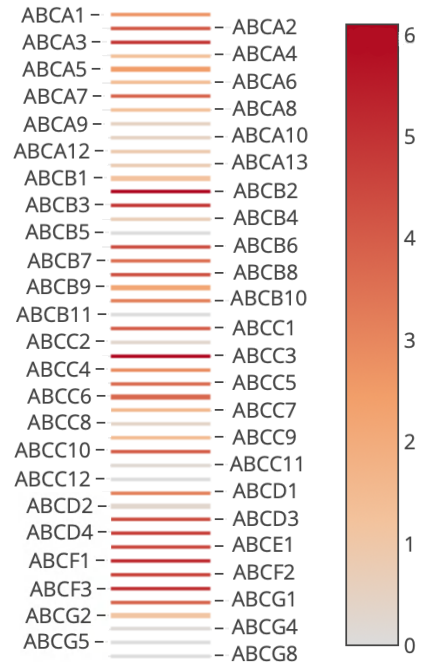

C

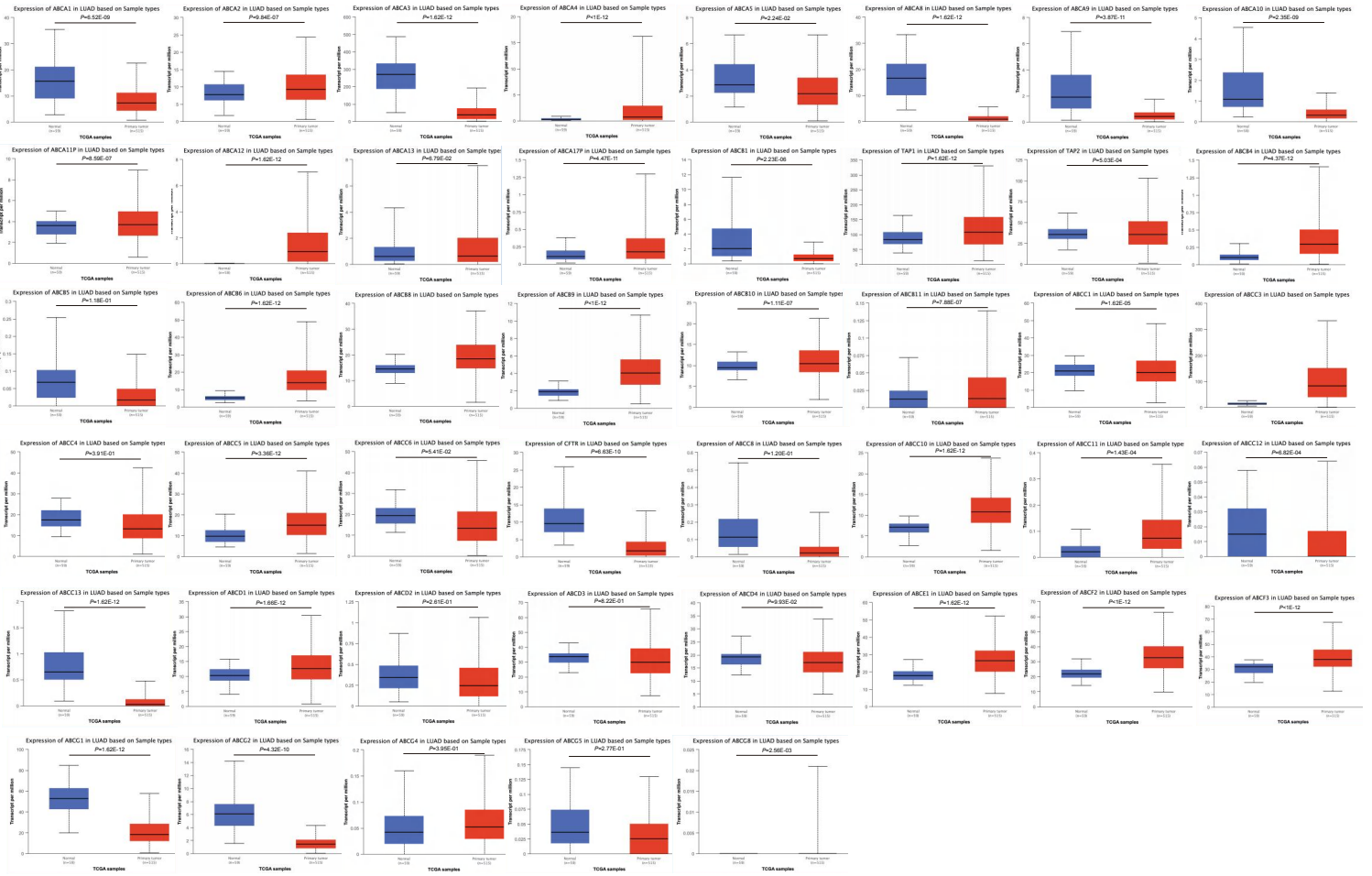

Supplement: Multimedia component 2 [file mmc2.pdf]

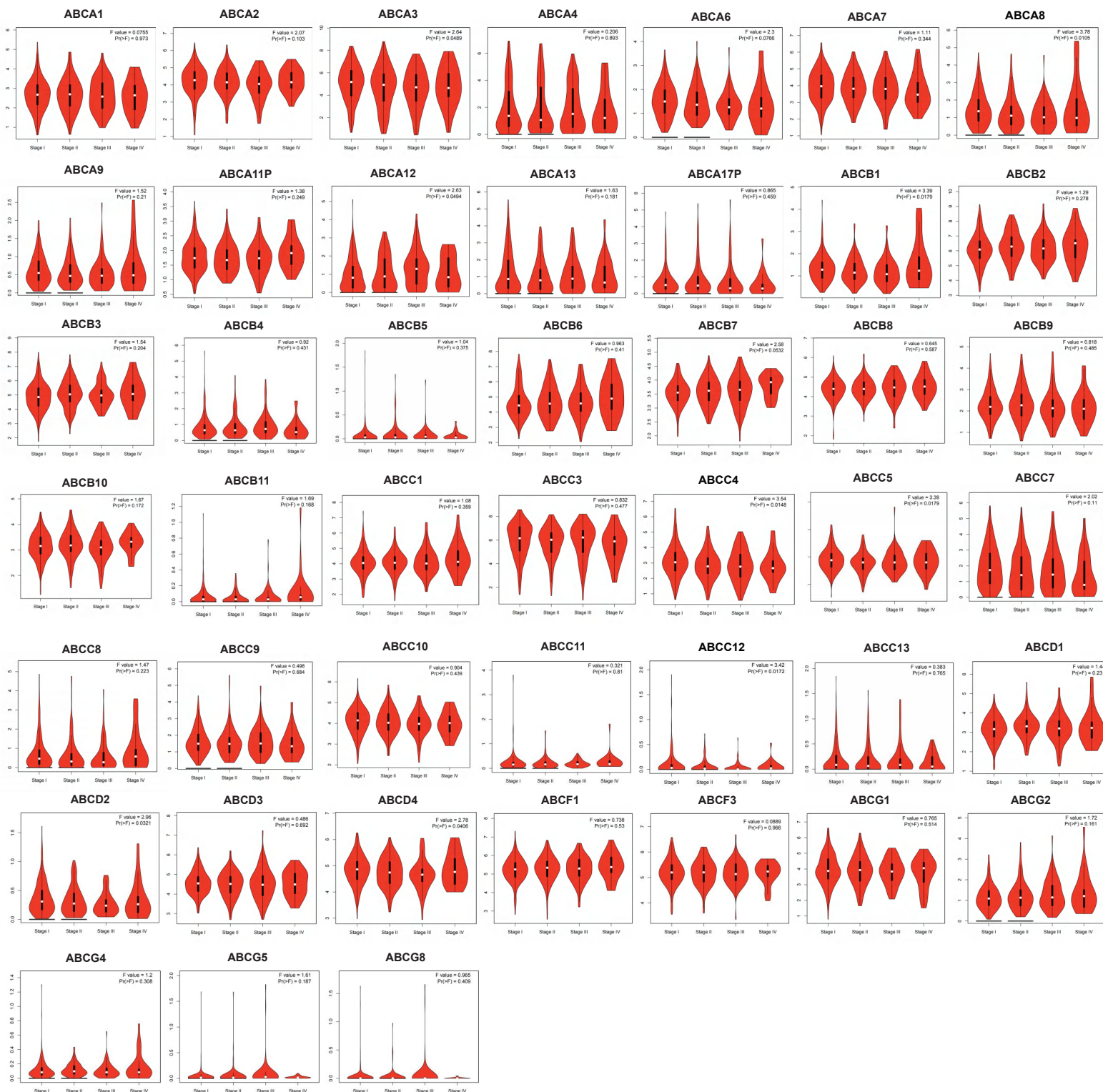

Supplement: Multimedia component 3 [file mmc3.pdf]

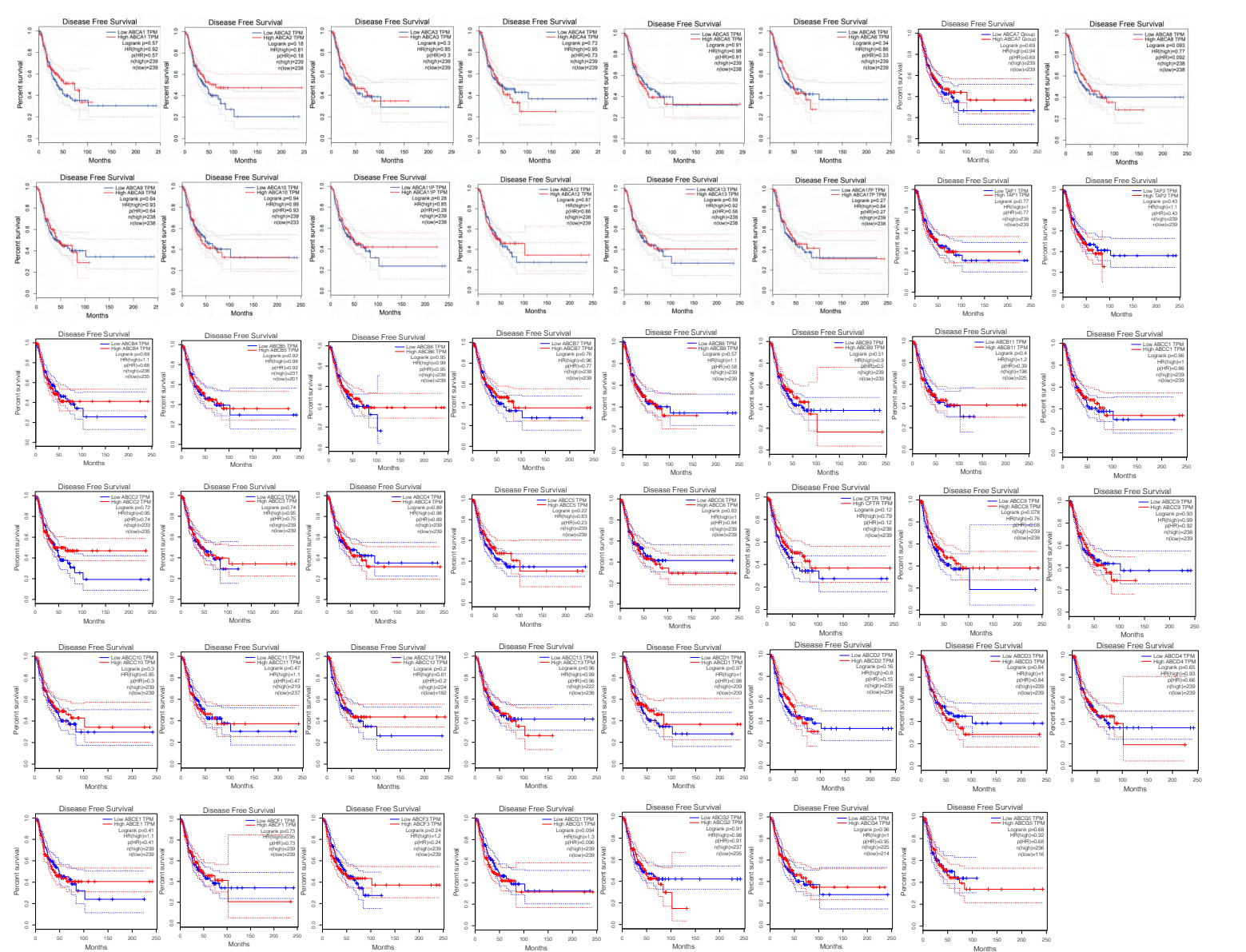

Supplement: Multimedia component 4 [file mmc4.pdf]

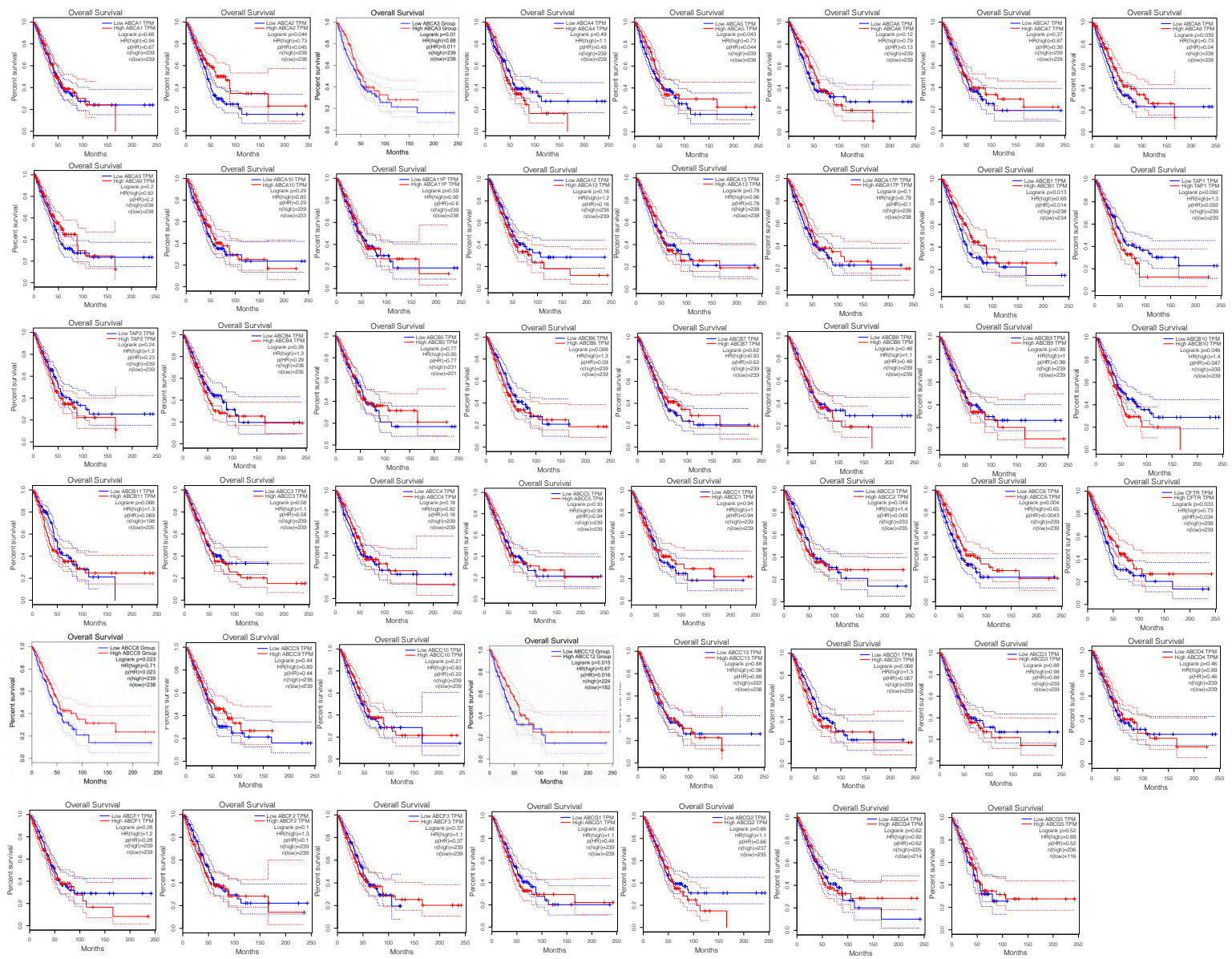

Supplement: Multimedia component 5 [file mmc5.pdf]

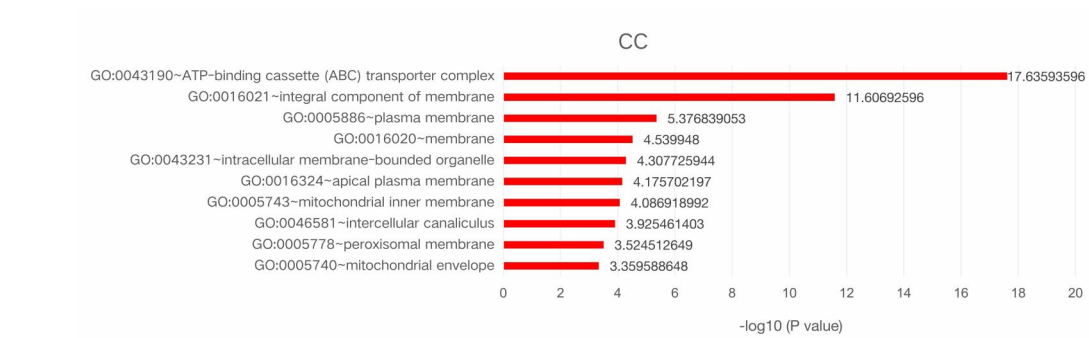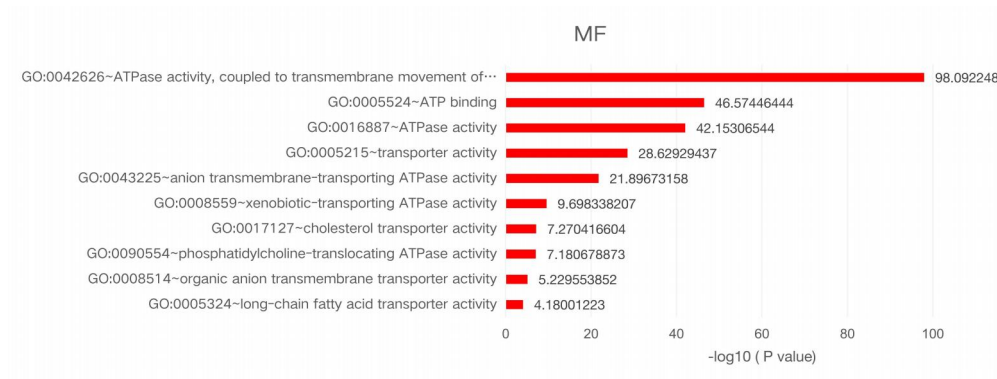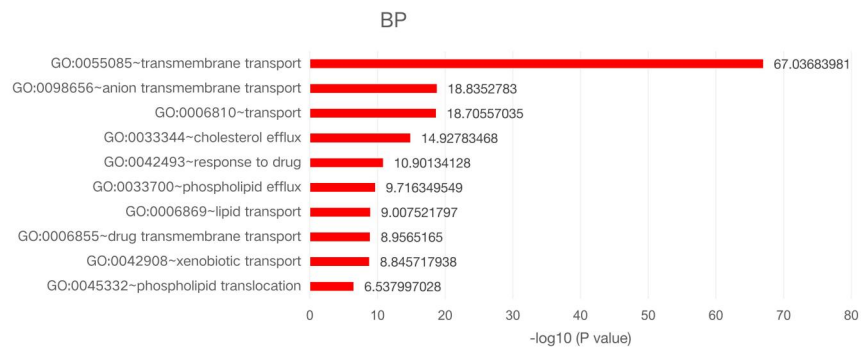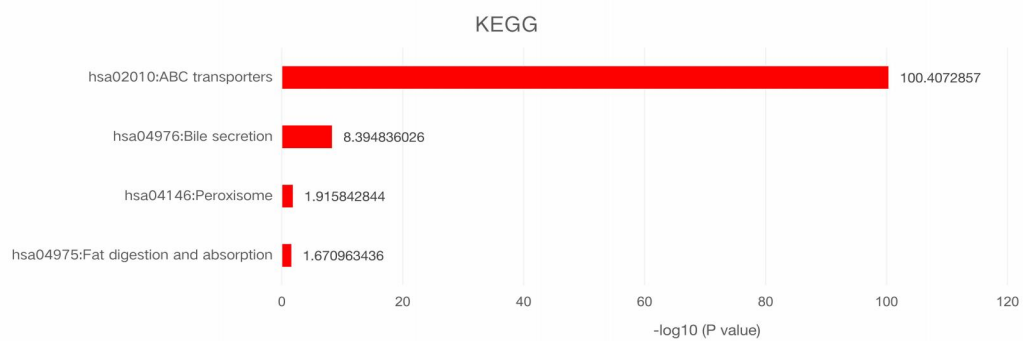

Supplement: Multimedia component 6 [file mmc6.pdf]

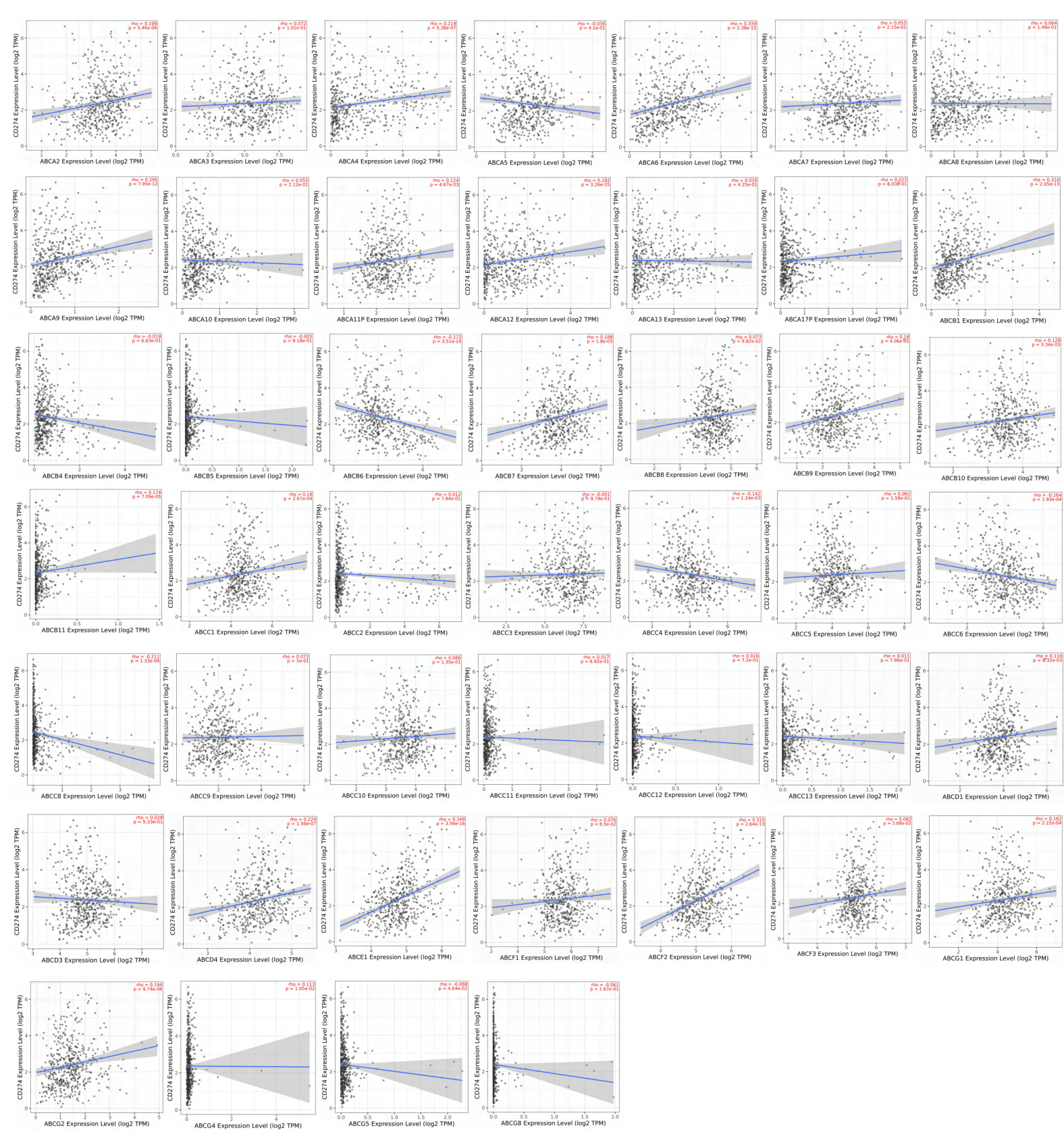

Supplement: Multimedia component 8 [file mmc8.pdf]

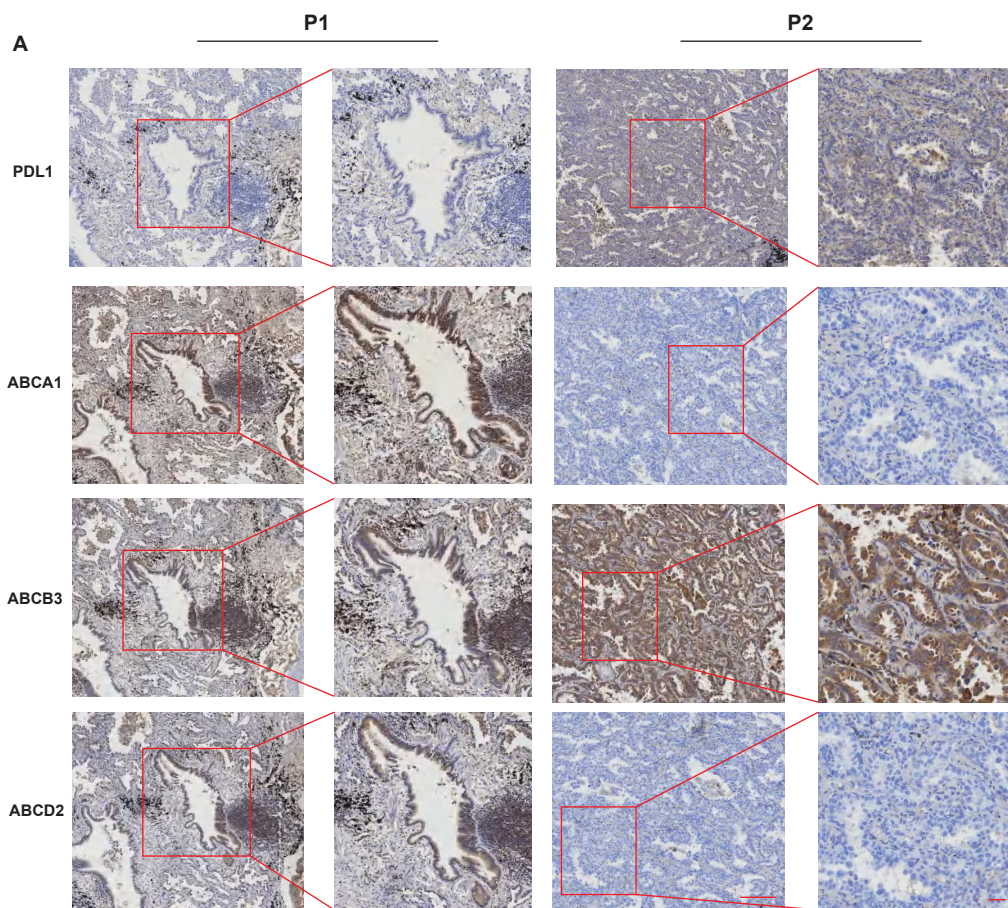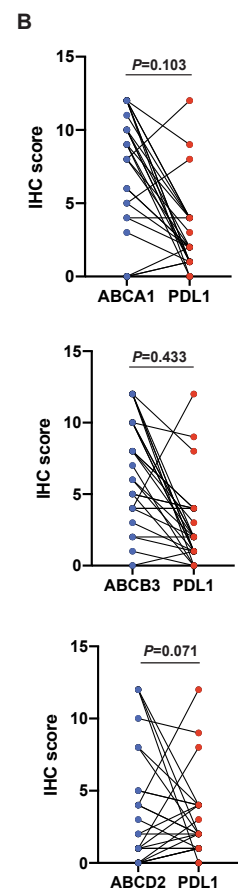

Supplement: Multimedia component 9 [file mmc9.pdf]
