# Supplementary material for: Promising prognostic value of ATP binding cassette transporters and their correlation with tumor-infiltrating immune cells in lung adenocarcinoma
Source: Genes Dis. 2023 Sep 14;11(5):101099. doi: 10.1016/j.gendis.2023.101099 (PMC11176628; doi:10.1016/j.gendis.2023.101099)
Supplement: Multimedia component 7 [file mmc7.pdf]

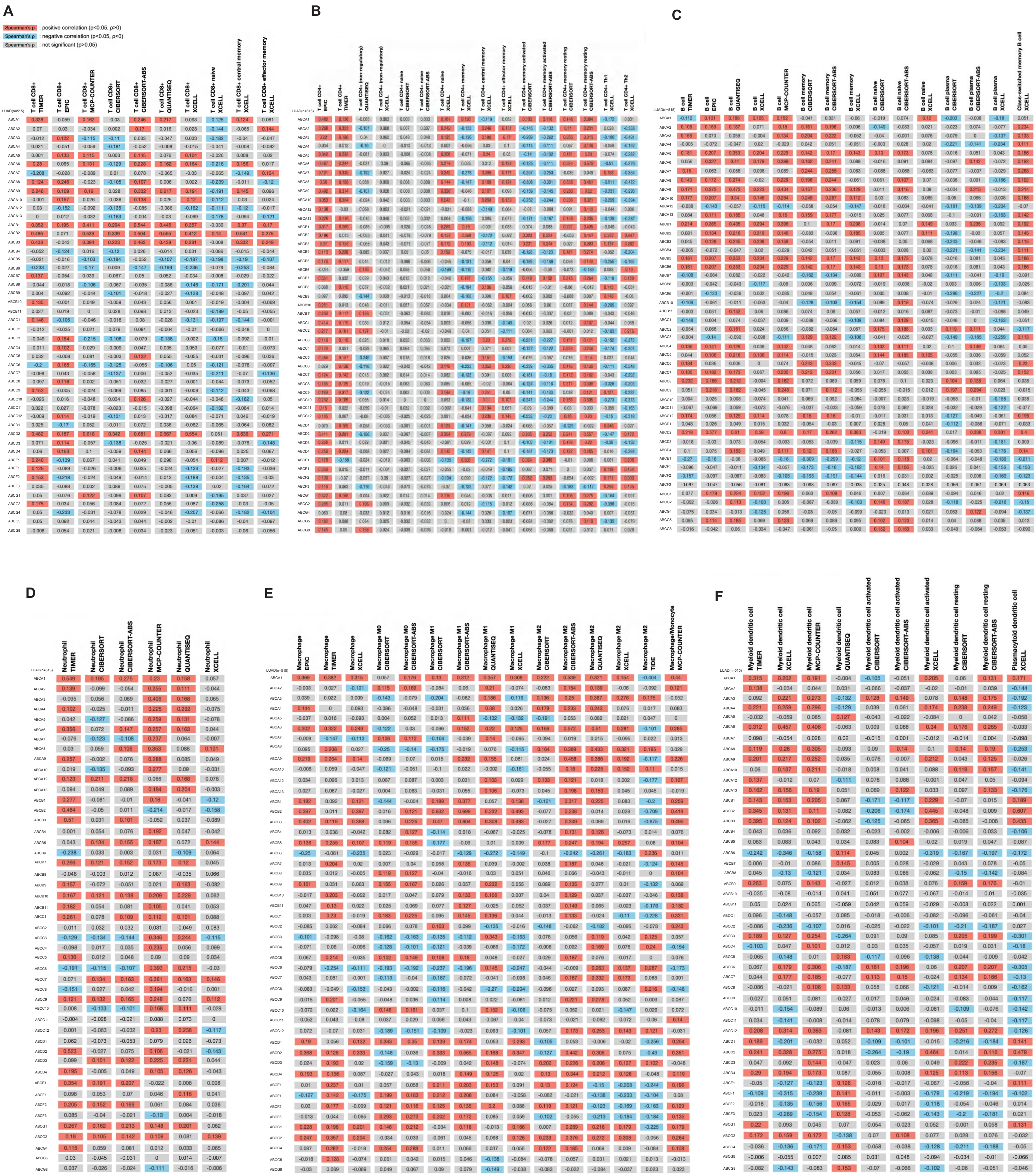

A
B
C
D
E
F
Spearman's ρ : positive correlation (p<0.05, ρ>0)
Spearman's ρ : negative correlation (p<0.05, ρ<0)
Spearman's ρ : not significant (p>0.05)
